# Evaluating the Role of the Jaw Thrust Maneuver During Tracheal Intubation in Reducing the Incidence of Postoperative Sore Throat: A Prospective Randomized Study

**DOI:** 10.31486/toj.24.0072

**Published:** 2025

**Authors:** Dipti Saxena, Arohi Rathore, Pallavi Jain, Anuj Jain, Swapnil Kumar Barasker

**Affiliations:** ^1^ Department of Anesthesiology, Sri Aurobindo Medical College and Post Graduate Institute, Indore, India; ^2^ Department of Anaesthesiology, All India Institute of Medical Science, Bhopal, India

**Keywords:** *Anesthesia–general*, *intubation*, *hoarseness*, *pharyngitis*, *postoperative period*

## Abstract

**Background:**

Endotracheal intubation is commonly associated with postoperative sore throat. We evaluated the effect of the jaw thrust maneuver on the incidence and severity of sore throat.

**Methods:**

A total of 110 female patients undergoing laparoscopic abdominal procedures were enrolled in the study, and 99 patients were included in the final analysis. The patients were randomized into 2 groups: the jaw thrust group (n=49) and the non–jaw thrust group (n=50). Sore throat monitoring and grading were performed at 0, 2, 4, 8, 12, and 24 hours postextubation.

**Results:**

The overall incidence of sore throat was higher in the non–jaw thrust group than in the jaw thrust group, with a maximum incidence at 4 hours, and the difference in incidence between the 2 groups was significant at the 4-, 8-, 12-, and 24-hour time points postextubation. However, no significant difference in sore throat severity was found between the non–jaw thrust and jaw thrust groups. The time of laryngoscopy was significantly less for patients in the jaw thrust group.

**Conclusion:**

In our population, the jaw thrust maneuver lowered the incidence but not the severity of sore throat during the initial 24 hours after extubation. The jaw thrust maneuver also significantly lowered laryngoscopy time.

## INTRODUCTION

The incidence of sore throat reported by patients undergoing surgery under general anesthesia with endotracheal intubation ranges from 10% to 62%.^1,2^ Sore throat is among the most common complaints that patients report within 24 hours after extubation.^3^ Laryngoscopy for intubation with endotracheal tubes causes minor soft tissue trauma that leads to sore throat. Prolonged intubation, high cuff pressure, female sex, smoking history, and use of a double lumen tube add to the risk of sore throat.^4^ Patient position during surgery—specifically supine to prone—is an independent factor for increased incidence of sore throat.^5^ The complaints of sore throat peak 2 to 6 hours postoperatively and generally decrease thereafter, but trauma can extend the time of discomfort.^6^

The jaw thrust maneuver is commonly used for airway management and is part of the basic training of an anesthesiologist. The jaw thrust maneuver prevents the tongue from falling backward by lifting it from the base, thereby improving upper airway patency,^7^ and also widens the orotracheal zone.^8^ Consequently, laryngoscopy should be easier to perform.

We hypothesized that the use of the jaw thrust maneuver during endotracheal intubation would decrease the incidence and severity of postoperative sore throat. For this study, we evaluated the effect specifically in female patients, as the incidence of postoperative sore throat is higher among females.^4^ Patient selection was further restricted to those undergoing only laparoscopic abdominal surgeries under general anesthesia with endotracheal intubation because patients were positioned supine during these surgeries.

## METHODS

Ethics approval was obtained from the institutional ethics committee (SAIMS/IEC/2021/25), and the clinical trial was registered with the Clinical Trial Registry of India (CTRI/2021/05/033390) before patients were enrolled. This prospective randomized trial was conducted at a single center in accordance with the Declaration of Helsinki 2013. The study period was June 2021 to January 2023.

After an explanation of the study was provided, informed written and verbal consent was obtained from all eligible patients. Female patients aged 20 to 60 years with American Society of Anesthesiologists (ASA) physical status I or II who were undergoing planned laparoscopic abdominal surgeries in the supine position were eligible. Patients with a recent history of sore throat or upper respiratory infection, steroid intake, and maxillofacial surgeries were excluded. Patients with a difficult airway or laryngoscopy, failed intubation, duration of laryngoscopy >30 seconds, surgeries extending beyond 2 hours, and reoperation during the observation time of 24 hours were also excluded.

The patients were randomized into 2 groups using computer-generated tables. The group allocation was revealed only to the anesthesiologist responsible for the jaw thrust maneuver.

The anesthesiologist responsible for data collection was unaware of the patient group assignments. A single anesthesiologist performed the intubation for all patients in the study. We attempted to use the technique defined by Park et al^9^ to blind the anesthesiologist responsible for intubation, but this procedure was ineffective.

### Procedure

Nil per oral guidelines as defined by ASA were followed. Patients were premedicated with intravenous (IV) midazolam (0.02 mg/kg) 30 minutes prior to the planned induction of anesthesia. On arrival of the patient in the operating room, ASA standards of monitoring were followed: electrocardiography, noninvasive blood pressure, pulse oximetry, end-tidal carbon dioxide (ETCO_2_), and train-of-four (TOF). After preoxygenating the patient for 3 minutes, IV fentanyl (2 μg/kg) was injected, followed by IV propofol (2.0 mg/kg). On confirming the patient's ability to ventilate on the mask, IV cisatracurium (0.2 mg/kg) was administered. After 3 minutes of administering cisatracurium, the TOF was checked, and if the recordings were absent, the anesthesiologist responsible for jaw thrust administered the procedure in the patients randomized to the jaw thrust group. Intubation followed.

The intubating anesthesiologist announced the Cormack-Lehane grade^10^ and percentage of glottic opening score^11^ prior to intubation. A Macintosh laryngoscope blade size 3 was used, with a high-volume, low-pressure sterile polyvinyl chloride endotracheal tube with an internal diameter of 6.5 mm or 7.0 mm as determined per the intubating anesthesiologist's assessment. Time of laryngoscopy was defined as the time from insertion of the laryngoscope blade to removal of the laryngoscope blade after insertion of the endotracheal tube.

The endotracheal tube cuff was inflated with air to a cuff pressure of approximately 20 cm of H_2_O according to endotracheal cuff pressure monitoring. After confirming bilateral air entry and ETCO_2_ waveform, the tube was fixed. For the maintenance of anesthesia, a mixture of oxygen and air (1:1) with isoflurane (1.2 minimum alveolar concentration) and cisatracurium maintenance (0.03 mg/kg) as intermittent bolus were used. IV ondansetron 4 mg was administered 30 minutes prior to the end of surgery. Port site subcutaneous infiltration with 0.5% bupivacaine was done before skin closure. Care was taken while suctioning the posterior pharyngeal wall to avoid trauma.

The patient was reversed with IV neostigmine (50 μg/kg) and glycopyrrolate (10 μg/kg) if TOF was <0.4 or with IV neostigmine (20 μg/kg) and glycopyrrolate (10 μg/kg) if TOF was 0.4 to 0.9. Inspiratory oxygen concentration was increased to 100%, and IV 2% lidocaine (preservative free, 1.5 mg/kg) was administered prior to extubation. Patients were extubated when they were breathing spontaneously and following commands.

For all patients, the same anesthesia protocol was used during the perioperative period. For postoperative analgesia, all patients received IV paracetamol 1 g every 8 hours. For patients who required rescue analgesia, IV tramadol 100 mg infusion over a half-hour period was used.

Sore throat monitoring and grading were performed at 0, 2, 4, 8, 12, and 24 hours postextubation. Sore throat was defined as continuous throat pain, and the grading scale defined by Knoll et al^12^ was used. At the 6 time points, patients were asked if they had a sore throat. If the answer was no, grade 0 was recorded. If the answer was yes, the patient's sore throat was graded as follows: (1) I=mild (pain with deglutition); (2) II=moderate (pain present constantly and increasing with deglutition); or (3) III=severe (pain interfering with eating and requiring analgesic medication).

Patients were also asked if they had any jaw discomfort at 0, 2, 4, 8, 12, and 24 hours postextubation. Jaw discomfort was defined as pain in or around the temporomandibular joint or pain when opening the mouth or chewing. The response was recorded in the form of yes or no.

### Sample Size

For calculating the sample size, the incidence of sore throat was assumed to be 65%.^2^ We hypothesized that the use of the jaw thrust maneuver would reduce the incidence of sore throat by 50%. For a power of 90%, β=0.01, and α=0.05, 96 patients (48 in each group) were required. To compensate for possible dropouts, we enrolled 110 patients (55 in each group).

### Statistical Analysis

SPSS Statistics version 20.0 (IBM Corporation) was used for statistical analysis. The normality of parameters such as age, weight, Mallampati grade, ASA physical status, and Cormack-Lehane grade was assessed using the Shapiro-Wilk test. Quantitative data are expressed as counts and percentages and as means ± standard deviation. Intergroup comparison of time of laryngoscopy and duration of surgery was done using an independent *t* test. The association of categorical variables such as incidence and severity of sore throat and jaw discomfort were analyzed using chi-square test or Fisher exact test. *P* value <0.05 was considered statistically significant.

## RESULTS

**Figure 1.**
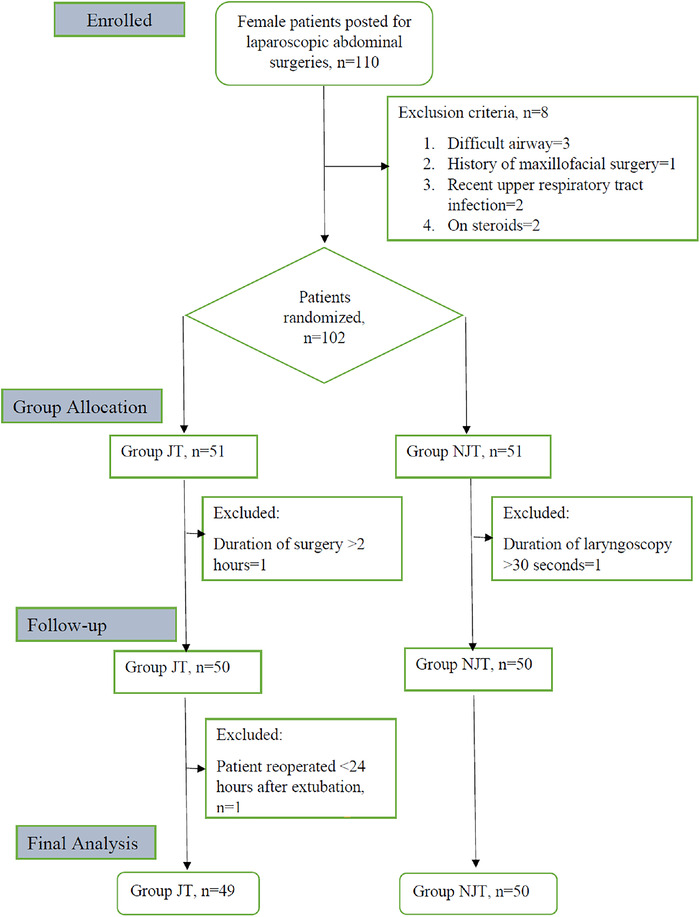
**Consolidated Standards of Reporting Trials (CONSORT) flow diagram.** JT, jaw thrust; NJT, non–jaw thrust.

A total of 110 patients were enrolled in the study. Eight patients were excluded prior to randomization. After group allocation, 3 patients were excluded, so 99 patients were included in the final analysis (Figure 1). All intubations were done in a single attempt.

Clinical characteristics by group are presented in Table 1. Time of laryngoscopy was significantly less in the jaw thrust group compared to the non–jaw thrust group. Heart rate and mean arterial pressure were recorded before and 2 minutes after laryngoscopy; no significant differences in either parameter at either time point were found between the groups.

**Table 1.** Clinical Characteristics by Group

Variable	Jaw Thrust Group, n=49	Non–Jaw Thrust Group, n=50	*P* Value
Age, years, mean ± SD	37.37 ± 13.48	37.45 ± 12.66	0.985
Weight, kg, mean ± SD	58.68 ± 11.74	58.10 ± 12.23	0.880
Body mass index, kg/m^2^, mean ± SD	25.16 ± 4.16	25.66 ± 3.90	0.697
Smoker			0.570
Yes	1 (2.0)	2 (4.0)	
No	48 (98.0)	48 (96.0)	
Mallampati grade			0.776
I	33 (67.3)	35 (70.0)	
II	16 (32.7)	15 (30.0)	
American Society of Anesthesiologists physical status			0.621
I	30 (61.2)	33 (66.0)	
II	19 (38.8)	17 (34.0)	
Time of laryngoscopy, seconds, mean ± SD	18.53 ± 7.46	23.45 ± 5.15	**0.021**
Cormack-Lehane grade			0.205
I	30 (61.2)	22 (44.0)	
IIa	16 (32.7)	22 (44.0)	
IIb	3 (6.1)	6 (12.0)	
Percentage of glottic opening score, mean ± SD	75 ± 17.68	67.5 ± 20.58	0.204
Duration of surgery, minutes, mean ± SD	69.79 ± 24.20	70.20 ± 21.39	0.956
Incidence of sore throat	20 (40.8)	33 (66.0)	**0.0208**
Heart rate, mean ± SD			
Before laryngoscopy	82.63 ± 8.254	82.35 ± 10.236	0.925
2 minutes after laryngoscopy	96.32 ± 8.334	93.85 ± 8.628	0.370
Arterial pressure, mean ± SD			
Before laryngoscopy	89.42 ± 7.221	87.50 ± 8.262	0.444
2 minutes after laryngoscopy	96.26 ± 6.306	96.15 ± 5.842	0.954
Jaw discomfort	5 (10.2)	3 (6.0)	0.204

Notes: Data are presented as n (%) unless otherwise indicated. Statistical significance is identified with bold.

**Figure 2.**
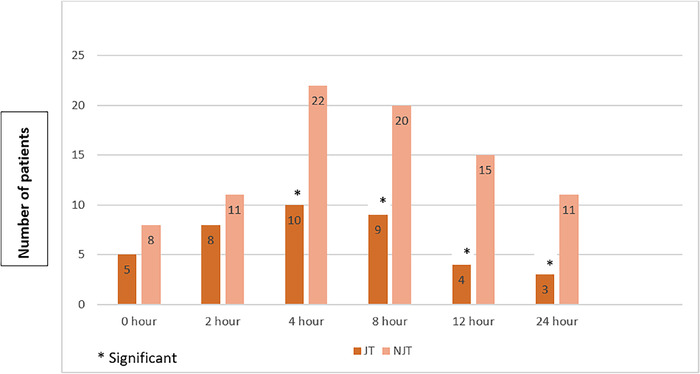
**Incidence of sore throat at postextubation time points by group.** JT, jaw thrust group; NJT, non–jaw thrust group.

The overall incidence of sore throat was 53.5% (53/99). The non–jaw thrust group had a significantly higher incidence of sore throat (66.0%) than the jaw thrust group (40.8%) (*P*=0.0208). At 4 (*P*=0.0218), 8 (*P*=0.032), 12 (*P*=0.0123), and 24 (*P*=0.0479) hours postextubation, the incidence of sore throat was significantly different between the 2 groups (Figure 2). Sore throat severity (grades II and III) was higher in the non–jaw thrust group, but no significant differences were found between groups at any of the postextubation time points (Table 2).

**Table 2.** Severity of Sore Throat at Postextubation Time Points by Group

Postextubation Time Point, hours	Sore Throat Grade	Jaw Thrust Group, n=49	Non–Jaw Thrust Group, n=50	*P* Value
0	I	4 (8.2)	6 (12.0)	0.639
	II	1 (2.0)	2 (4.0)	
	III	0	0	
2	I	6 (12.2)	8 (16.0)	0.677
	II	2 (4.1)	3 (6.0)	
	III	0	0	
4	I	6 (12.2)	13 (26.0)	0.446
	II	3 (6.1)	8 (16.0)	
	III	1 (2.0)	1 (2.0)	
8	I	8 (16.3)	13 (26.0)	0.377
	II	1 (2.0)	7 (14.0)	
	III	0	0	
12	I	3 (6.1)	11 (22.0)	0.567
	II	1 (2.0)	4 (8.0)	
	III	0	0	
24	I	2 (4.1)	8 (16.0)	0.606
	II	1 (2.0)	3 (6.0)	
	III	0	0	

Note: Data are presented as n (%).

The jaw of 1 patient in the jaw thrust group was dislocated bilaterally and repositioned intraoperatively. The overall incidence of jaw discomfort was 8.1% (8/99), with 10.2% in the jaw thrust group and 6.0% in the non–jaw thrust group reporting jaw discomfort. The difference between groups was not statistically significant (*P*=0.204).

## DISCUSSION

This study demonstrated a lower incidence of sore throat among female patients undergoing laparoscopic surgery when the jaw thrust maneuver was used during endotracheal intubation. Although the difference in incidence between groups was statistically significant, the difference did not meet our hypothesis of a 50% reduction in the jaw thrust group (66.0% vs 40.8%, a 38.2% decrease). Similarly, while our data supported our hypothesis that sore throat severity would be reduced in the jaw thrust group, the differences between groups were not significant. Time of laryngoscopy was reduced in patients who received the jaw thrust maneuver.

The jaw thrust maneuver is widely used to maintain the airway by preventing tongue falls in unconscious or sedated patients. The maneuver lifts the tongue from its base and increases the space of the laryngeal inlet.^6,7^ During intubation, the pharyngeal mucosa is traumatized by the laryngoscopy blade and the tip of the endotracheal tube during insertion or suctioning. This microtrauma manifests as sore throat during the postoperative period.^13^ Any measure that reduces this trauma will help reduce the incidence of sore throat.

In our literature search, we found 2 studies that evaluated the use of the jaw thrust maneuver to prevent sore throat.^9,13^ In contrast to our study, Park et al evaluated patients of both sexes undergoing thoracic surgeries in the lateral decubitus position with double lumen tube insertion, and they included surgeries extending beyond 2 hours.^9^ The overall incidence of sore throat in our study (53.5%) was similar to the incidence in the Park et al study (50.0%). Park et al also found that the jaw thrust maneuver resulted in a significant reduction in the severity of sore throat, while our study failed to show any such difference.^9^

In the other study, Huh et al showed that both sore throat and hoarseness incidence and severity were reduced in the postoperative period after the jaw thrust maneuver during endotracheal intubation,^13^ whereas we found a significant difference only in incidence and not in severity of sore throat. Similar to the Park et al study,^9^ the Huh et al study^13^ included patients of both sexes and in lateral decubitus positions, but the patients in the Huh et al study underwent orthopedic procedures instead of thoracic procedures. Huh et al evaluated jaw discomfort and did not find any difference between the jaw thrust and control groups.^13^ The results were similar in our study, but a complication was reported in our study. A patient's jaw was dislocated, immediate consultation was sought from the maxillofacial surgeon, and the jaw was repositioned intraoperatively. The patient reported no jaw discomfort during the postoperative period.

Jaw dislocation is a known complication of the jaw thrust maneuver,^14^ and the jaw thrust maneuver applied during mask ventilation resulted in significant jaw discomfort in an investigation by Brimacombe et al.^15^ The duration of mask ventilation is usually longer than that of laryngoscopy, which may explain this difference.

Similar to the Park et al and Huh et al studies,^9,13^ we observed no difference in the Cormack-Lehane grade and percentage of glottic opening score for patients in the jaw thrust group compared to those in the non–jaw thrust group. A lower Cormack-Lehane grade and higher percentage of glottic opening score are associated with a lower incidence of sore throat,^16^ as the need for additional manipulation is minimized and thus the chances of trauma to the airway are also minimized.

The use of the jaw thrust maneuver to improve ventilation has been shown to provoke a sympathetic response, evident with raised blood pressure and heart rate.^17^ However, our study showed no difference in either parameter between the groups. This finding could be explained by the use of noninvasive blood pressure monitoring in our study and by the timing of the jaw thrust maneuver. The jaw thrust maneuver was coincident with laryngoscopy, thus masking the sympathetic effect.

Prolonged laryngoscopy during endotracheal intubation is associated with increased incidence of sore throat.^4^ Any effort to lower the time of laryngoscopy might help in decreasing the incidence of sore throat. We observed a significantly (*P*=0.021) lower time of laryngoscopy in patients in the jaw thrust group. The shorter time of laryngoscopy could be explained by laryngeal inlet widening by the jaw thrust maneuver,^7^ but this effect was not observed in the Park et al study,^9^ because the jaw thrust maneuver was used for insertion of a double lumen tube instead of an endotracheal tube.

A wide array of drugs—including lidocaine, magnesium, ketamine, corticosteroids, and nonsteroidal anti-inflammatory drugs—with varied mechanisms of action and administered via various routes including topical, nebulization, and IV have been used to try to reduce the incidence and severity of sore throat.^18^ Nonpharmacologic measures, such as video laryngoscopes^16^ and thermal softening of endotracheal tubes,^19^ have also been used, but the jaw thrust maneuver appears to decrease the incidence of sore throat without the need for extra resources in resource-limited settings.

### Limitations

The current study has multiple limitations. First, the study population included female patients only. Second, the long-term effects of the jaw thrust maneuver were not evaluated; patients were observed for only 24 hours postextubation. Third, patients with difficult airways were not included in the analysis, so the role of the jaw thrust maneuver in such patients cannot be ascertained. A fourth limitation is the lack of blinding of the intubating anesthesiologist. Because assessment of Cormack-Lehane grade and percentage of glottic opening score are operator dependent, the absence of blinding could introduce potential bias. However, this risk was mitigated by using consistent perioperative anesthesia protocols, assigning a single anesthesiologist for all intubations to eliminate interoperator variability, and using objective metrics such as time of laryngoscopy. Furthermore, sore throat grading data were collected by a different anesthesiologist who was blinded to group allocation.

## CONCLUSION

This study suggests that the jaw thrust maneuver is a simple technique that can help reduce the incidence but not the severity of sore throat and can potentially reduce the time of laryngoscopy.

## Notes

*This article meets the Accreditation Council for Graduate Medical Education and the American Board of Medical Specialties Maintenance of Certification competencies for Patient Care and Medical Knowledge.*
